# Refractory Tophaceous Gout With Psoriatic Arthritis: A Case Report

**DOI:** 10.7759/cureus.74079

**Published:** 2024-11-20

**Authors:** Rui Coelho, Isabel Fonseca Silva, Sara Xavier Pires, Tomás Fonseca

**Affiliations:** 1 Internal Medicine, Unidade Local de Saúde de Santo António, Porto, PRT

**Keywords:** anakinra treatment, anti-il-1 treatment, psoriatic arthiritis, refractory gout, tophaceous gout

## Abstract

Although gout is a common intermittent crystalline arthropathy, tophaceous gout is a rare condition. Flares of this disease are usually treated with anti-inflammatory drugs followed by control of serum uric acid levels. We present a refractory, severe, tophaceous gout overlapping with psoriatic arthritis, presenting with a hyper-inflamed phenotype resistant to conventional anti-inflammatory and hypouricemic agents. Control of the disease was only achieved after the introduction of IL-1-blocking therapy. This case highlights the need to control inflammation as the first step in gout treatment and underscores the increasing need for personalized therapies in more complex patients.

## Introduction

Gout is a common inflammatory disease caused by the precipitation of monosodium urate crystals (MSU). Uric acid, as a damage-associated molecular pattern (DAMP), activates the NLRP3 inflammasome, which increases interleukin-1 (IL-1) and interleukin-18 (IL-18) levels. These cytokines activate the granuloma response, leading to tophi formation and therefore causing erosive arthropathy [1-3].

As an intermittent disease, the treatment of gout aims to prevent and control flares, focusing on decreasing serum uric acid (SUA) using urate-lowering therapies (ULT) and managing comorbidities such as psoriasis. In tophaceous gout, an SUA level under 5 mg/dL is recommended. Gout flares induced by ULT are frequent, and anti-inflammatory prophylaxis should be used in the first six months of therapy [2]. Despite optimized therapy, some patients develop refractory gout, which has no precise definition [4,5], and therefore there is no definitive approach. In addition to new and effective hypouricemic drugs, in difficult-to-treat and refractory inflammatory patients, some studies have shown the benefits of the use of IL-1 blocking therapy [6,7].

We present a case of refractory tophaceous gout arthritis with multiple comorbidities and cumulative damage, in which control was only achievable after IL-1 blocking therapy. The authors aim to report an extremely refractory disease and emphasize the search for underlying diseases, such as psoriatic arthritis (PsA), which can contribute to a difficult-to-treat hyper-inflamed phenotype.

## Case presentation

**Figure 1:**
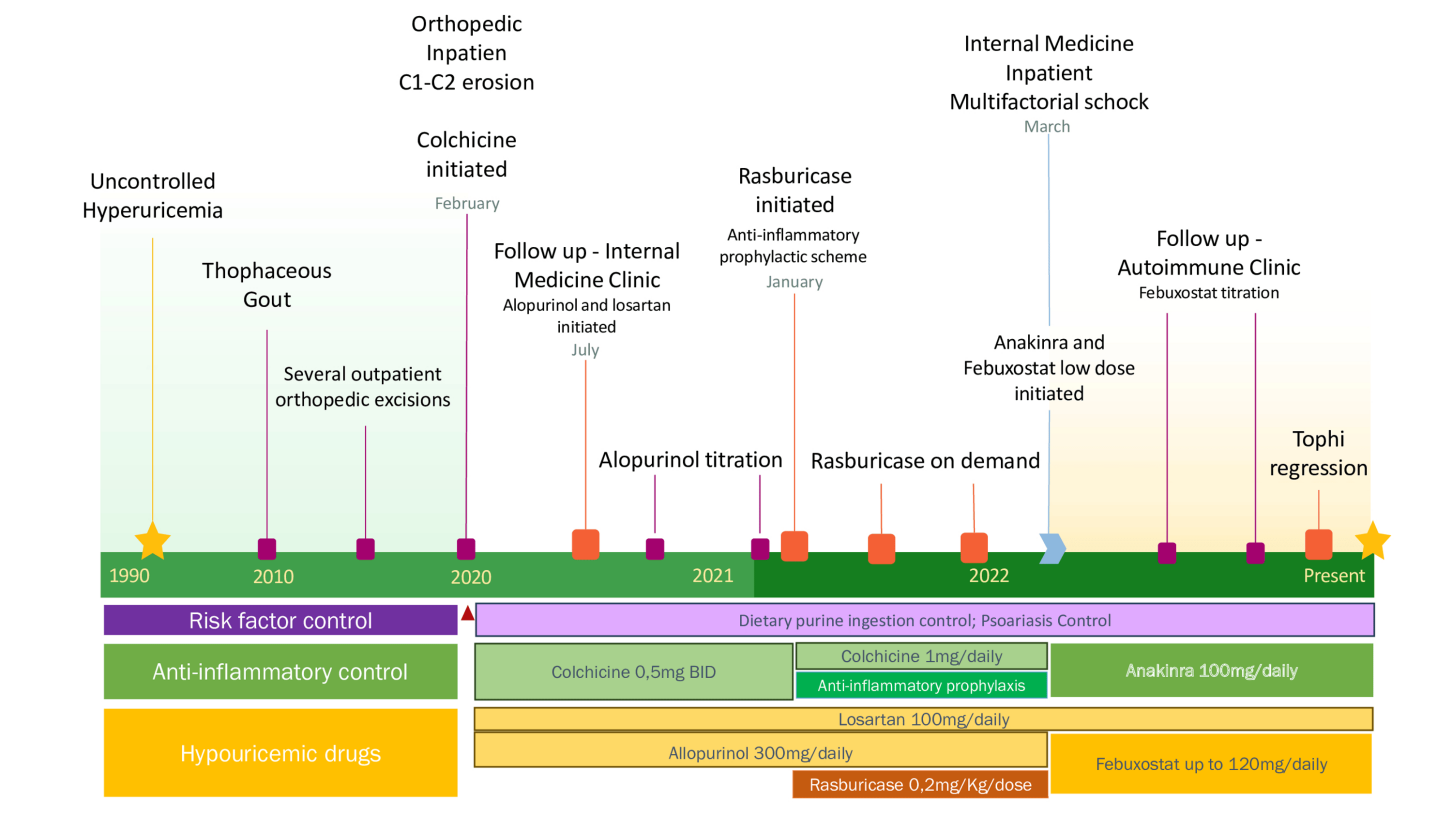
Timeline and evolution of patient's gout and treatments

We present a 63-year-old man with 30 years of untreated hyperuricemia and tophaceous gout, hypertension, hypercholesterolemia, ischemic cardiopathy, chronic kidney disease (3a KDIGO staging), vulgar psoriasis (PsO), and chronic alcohol consumption, without liver disease. The authors suggest following the patient's gout evolution along with Figure 1. Erosive polyarthritis and tophaceous gout have been present since 2010, initially documented in anterior legs and knees with later progression to upper limbs, with joints, skin, and cartilaginous tissue depositions. Several local orthopedic excisions of tophi were performed, with no proper medical treatment for gout, with SUA persistently above 10 mg/dL. History of cutaneous adverse reaction to febuxostat.

**Figure 2:**
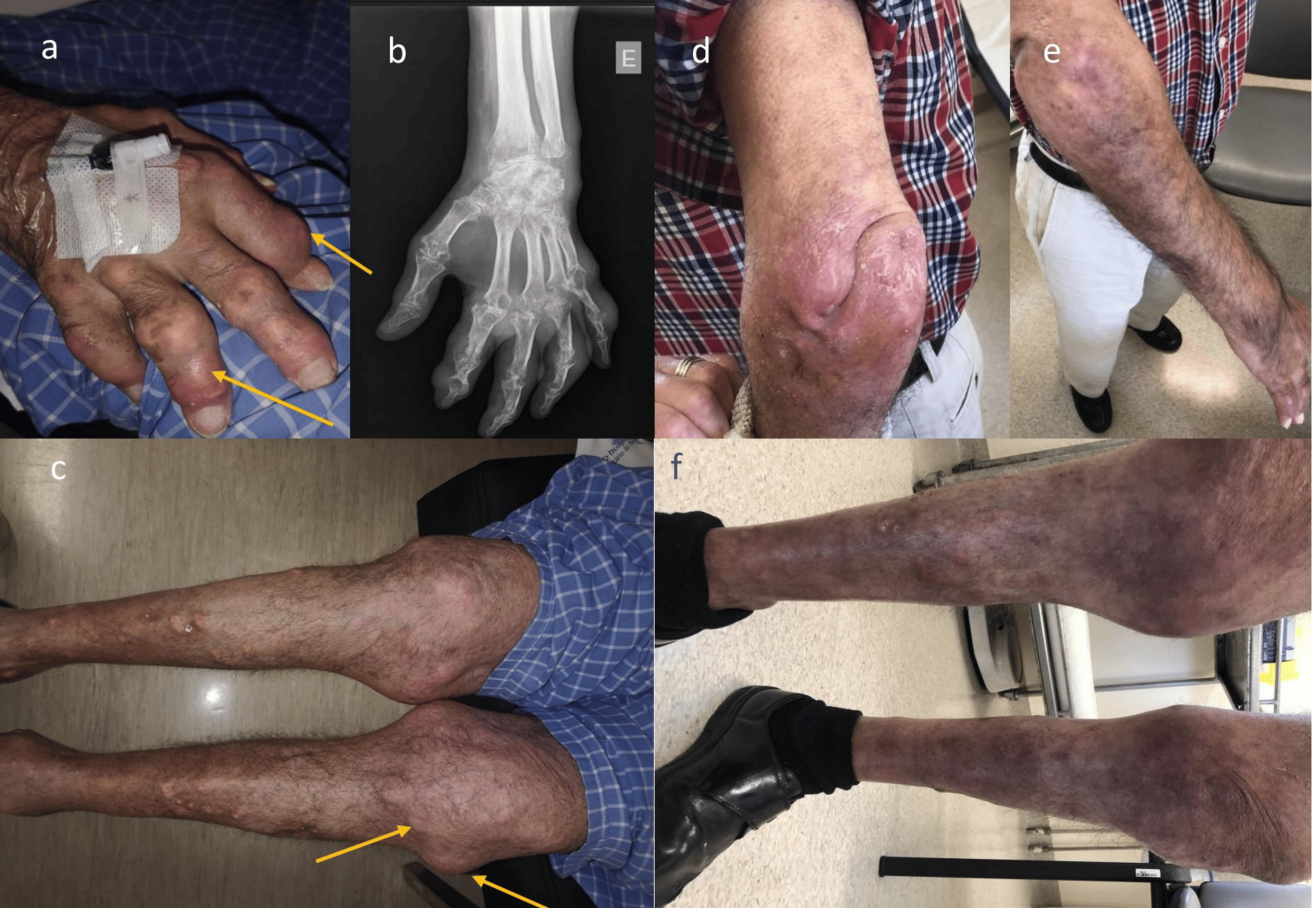
Patients interphalangeal, knees, and elbow joints before and after treatment a: Inter-phalangeal joints with tophaceous gout flare before treatment (June/2022); b: signs of articular damage after long-term non-controlled disease (June/2022); c: knee joints with tophaceous gout and articular damage before treatment - yellow arrow (June/2022); d and e: left (d) and right (e) elbow joints after treatment with no signs of tuphi or exsudative lesions (July/2023); f: knee joints after treatment with no signs of active gout flare and no tophi (July/2023)

In February 2020, a severe erosive arthritis of C1-C2 led to bulbar commitment, and an emergency arthrodesis was successfully performed, followed by a long-term stay in the orthopedics ward. After surgery, he began treatment for a gout flare with colchicine 0.5 mg twice daily. Five months later he was evaluated in the internal medicine outpatient clinic, with several disabling tophaceous lesions (Figure 2). ULT was initiated with allopurinol 100 mg/day, along with an antihypertensive drug switch to Losartan, dietary purine restriction (alcohol consumption was stopped), PsO treatment, and proper rehabilitation, which required orthoses to several joints. Prosthetic surgery was denied due to the risk of joint disruption and infection.

During follow-up visits, allopurinol was titrated up to 300 mg/day, without clinical benefit and with intolerance to higher dosages. Rasburicase was started at 0.2 mg/kg/dose. After a tumultuous first cycle with a severe provoked-gout flare, the authors added an intensive anti-inflammatory prophylaxis scheme with colchicine, high-dose prednisolone, and indomethacin. The treatment allowed partial control of the disease for six months, with persistently low SUA (under 5 mg/dL). Nevertheless, the patient still had sustained polyarthritis with purulent and hematic exudative tophi.

In March 2022, the patient was admitted to the emergency department due to multifactorial shock due to infection of the ulcerative elbow tophi and acute bleeding, starting antibiotics and supportive care. After the resolution of the critical illness, treatment failure was assumed, and the case was discussed in a multidisciplinary manner. Considering the presence of PsO, cervical axial arthritis in the past, and refractory peripheral small joints polyarthritis, it was assumed the diagnosis of PsA overlapped with tophaceous gout. The HLA-genetic analysis identified the PsA risk locus HLA-C*06:02. Anakinra 100 mg/day was initiated, and after arthritis control, the patient started low-dose febuxostat. He achieved rapid disease control, with no adverse events, and was discharged shortly thereafter.

Follow-up in the outpatient clinic

One month after discharge, he had no flares or need for non-steroidal anti-inflammatory drugs (NSAIDs) and presented without arthritis or exudative lesions. However, he still presented multiple tophi, elevated inflammatory markers, and high SUA (Table 1). As a treat-to-target strategy, febuxostat was slowly titrated up to 120 mg/day in subsequent visits.

**Table 1:** Laboratory results and tophi progression registered in the outpatient clinic follow-up after initiating anakinra and febuxostat titration

Data	Seric uric acid (normal range: 3.4-7.0 mg/dL)	Erythrocyte sedimentation rate (ERS) (normal range: 0-14 mm)	C-reactive protein (normal range: 0.0-5.0 mg/L)	
June 29, 2022	7.2 mg/dL	82 mm	44 mg/L	
February 15, 2023	6.2 mg/dL	51 mm	111 mg/L	
July 12, 2023	6.1 mg/dL	15 mm	10 mg/L	

After two years of continuous treatment with febuxostat and anakinra, the patient remains in prolonged clinical remission, without signs of arthritis or psoriatic lesions, and with resolution of tophi (Figure 2). Although SUA was not reduced to targeted (5 mg/dL), systemic inflammation is controlled (Table 1). He reports the best quality of life in years, with full autonomy (no longer requiring orthoses), and appears to be on an optimal treatment strategy.

## Discussion

Gout is an inflammatory arthropathy whose management consists of two simultaneous approaches: controlling inflammatory activity and articular damage and preventing new flares by lowering SUA. This case illustrates a long-term and untreated hyperuricemia, with refractory tophaceous gout in a patient with significant comorbidities.

At first, undertreatment was assumed, and the patient started on standard-of-care therapies. However, the emerging side effects, namely ULT and NSAID overuse, along with poor disease control, led to alternative treatment schemes.

Although some reports advocate the use of dual xanthine oxidase inhibitors to address persistently high SUA, the patient had high cardiovascular risk and a prior adverse cutaneous reaction to febuxostat. For these reasons, rasburicase was started, and SUA was successfully lowered. Nonetheless, the patient still presented flares, requiring intensive anti-inflammatory prophylaxis, which presents several complications in long-term control and, in this case, also led to the loss of therapeutic adherence.

Despite gout treatment, the patient did not experience tophi reduction, pain relief, or systemic inflammation control, and many complications emerged. Some clinical aspects suggested overlap with PsA: (1) the personal history of PsO; (2) axial involvement with thickening of a transversal ligament on MRI and C1-C2 arthritis and odontoid erosion, which are rare complications in gout more often reported in PsA [3,8]; (3) symmetrical, small joint, and peripheral arthritis not fully responsive to gout treatment; (4) presence of HLA-C*06:02. Although gout overlapped with PsA has been assumed, also known as "Psout," authors recognize that the use of dual-energy CT (DECT) or joint ultrasound at the time could enhance the confidence of the diagnosis [9].

Although anti-TNF-Alfa, interleukin-17 (IL-17), and interleukin-23 (IL-23) blockade therapies are the biologic disease-modifying anti-rheumatic drugs (bDMARDs) approved for PsA, some reports emphasize up-regulation of IL-1 in the skin and synovium of patients with psoriasis and PsA [10] and therefore Anakinra was used in small cohorts to treat PsA with success [10,11]. IL-1 blocking agents have also been used off-label with success in refractory gout cases to control flare episodes [6,7,12].

Anakinra allowed control of the patient's hyperinflammatory phenotype, enabling the introduction of a ULT. Despite rasburicase's effectiveness, its on-demand regimen posed a problem for this patient, and this extremely difficult-to-treat gout demanded continuous ULT therapy. Although previously reported cutaneous reactions, authors chose to initiate febuxostat, a powerful ULT with intrinsic anti-inflammatory effect [13], in the inpatient supervised environment with a step-up approach (as shown in Figure 1), with good tolerance and results.

## Conclusions

Clear definitions and treatment goals for difficult-to-treat gout are still lacking. These rare and severe patients demand quicker introduction of personalized therapies, like anti-IL1. The authors emphasize the pursuit of this unmet need in this clinical case. Controlling the inflammatory response was the only way to allow the introduction of second-line hypouricemic agents. Anakinra proved effective in refractory gout flares and, combined with a step-up febuxostat approach, allowed for tophi resolution and disease control. This case highlights the importance of controlling inflammatory response since it can perpetuate disease activity even with optimized hypouricemic agents. This paper also underscores the importance of investigating comorbidities and alternative rheumatologic diagnoses, especially in refractory cases that can be challenging to diagnose for the untrained clinician.
